# CFD simulation of water vapour condensation in the presence of non-condensable gas in vertical cylindrical condensers

**DOI:** 10.1016/j.ijheatmasstransfer.2012.10.051

**Published:** 2013-02

**Authors:** Jun-De Li

**Affiliations:** School of Engineering and Science, Victoria University, P.O. Box 14428, MC 8001, Melbourne, Australia

**Keywords:** Vapour condensation, Non-condensable gas, Heat and mass transfer, CFD

## Abstract

This paper presents the simulation of the condensation of water vapour in the presence of non-condensable gas using computational fluid dynamics (CFD) for turbulent flows in a vertical cylindrical condenser tube. The simulation accounts for the turbulent flow of the gas mixture, the condenser wall and the turbulent flow of the coolant in the annular channel with no assumptions of constant wall temperature or heat flux. The condensate film is assumed to occupy a negligible volume and its effect on the condensation of the water vapour has been taken into account by imposing a set of boundary conditions. A new strategy is used to overcome the limitation of the currently available commercial CFD package to solve the simultaneous simulation of flows involving multispecies and fluids of gas and liquid in separate channels. The results from the CFD simulations are compared with the experimental results from the literature for the condensation of water vapour with air as the non-condensable gas and for inlet mass fraction of the water vapour from 0.66 to 0.98. The CFD simulation results in general agree well with the directly measured quantities and it is found that the variation of heat flux in the condenser tube is more complex than a simple polynomial curve fit. The CFD results also show that, at least for flows involving high water vapour content, the axial velocity of the gas mixture at the interface between the gas mixture and the condensate film is in general not small and cannot be neglected.

## Nomenclature

*C_p_*: specific heat (kJ/(kg K))
*r*: radial coordinate (m)
*x*: axial coordinate (m)
*D*: diffusivity (m^2^/s)
*g*: gravity acceleration (m/s^2^)
*h*: specific enthalpy (J/kg)
*h_fg,i_*: latent heat of water vapour at the interface (kJ/kg)
*L*: length of the pipe (m)
$\overset{˙}{m}$: mass flow rate (kg/s)
*P*: pressure (kPa)
$q^{\prime \prime }$: heat flux (kW/m^2^)
$\overset{˙}{Q}$: sensible heat (kW)
*Re*: Reynolds number
*Sc*: Schmidt number
*r_i_*: tube inner radius (m)
*r_o_*: condenser tube outer radius (m)
*T*: temperature (°C)
*U*: axial mean velocity (m/s)
*V*: radial mean velocity (m/s)
*u*: velocity (m/s)
*y*: lateral position (m)
*E*: internal energy (J/kg)
*R*: universal gas constant (=8.314 J/(mol K))
*M*: molar mass
*Y*: mass fraction
*S*: source terms
*J*: mass flux of species (J/m^2^ s)

**Greek symbols**
*δ*: thickness of condensate film (*m*)
*ɛ*: energy dissipation rate (m)
*κ*: kinetic energy (J/kg)
*μ*: dynamic viscosity (kg/(ms))
*ρ*: density (kg/m^3^)
*α*: under-relaxation factor
*τ_g_*: interfacial shear stress (N/m^2^)
*λ*: thermal conductivity (W/(mK))

**Subscripts**
*f*, *i*: film interface
*f*: film
*l*: liquid
*x*: axial direction
*v*: vapour
*av*: air–vapour
*w*: water
*0*: inlet
*i*: index for species
*T*: temperature
*m*: mass

## 1 Introduction

Condensation of water vapour in the presence of non-condensable gases has many applications such as air conditioning, electricity generation, refrigeration, reactor safety, aerospace, desalination and some heat exchangers. However, a detailed understanding and our capability of predicting it, especially in cases of high mass fraction of water vapour, are still lacking. As a result, we need to enhance our understanding on the physics of water vapour condensation in the presence of non-condensable gases and to develop techniques to predict the heat and mass transfer involved numerically for industrial applications.

The analysis by the heat and mass transfer analogy in situations with water vapour condensation in the presence of non-condensable gases has been described by many researchers. Colburn and Hougen [1] were the first to develop a theory for condensation mass transfer which was controlled by the mass concentration gradient through the non-condensable layer. They described the heat transfer process as the sum of sensible heat and latent heat flows. Dehbi and Guentay [2] derived a theoretical prediction of heat and mass transfer in a vertical tube condenser from steam and non-condensable gas mixture. An algebraic equation for the film thickness was derived. The mass and heat transfer analogy was invoked to deduce the condensation rate. Munoz-cobo et al. [3] developed a theory for turbulent vapour condensation in vertical tubes when non-condensable gases are present and the condensate film thickness was calculated using an approximate method. Che, Da and Zhuang [4] used the method of Colburn and Hougen [1] to analyse the heat and mass transfer process for the condensation of water vapour from moist air in a tube. They conducted experiments and found that the convection–condensation heat transfer coefficient is 1.5–2 times higher than that of forced convection without condensation. There have been several experiments performed to study condensation of vapour–gas mixture in vertical tubes. Siddique [5], Kuhn [6] and Kuhn et al. [7] studied steam condensation in the presence of air flowing downwards in vertical tubes and cold water flowing upwards inside cooling jackets.

Many of the theoretical predictions of vapour condensation and heat transfer in the presence of non-condensable gas have focussed on the gas and vapour mixture. The cooling of the gas–vapour mixture is normally calculated by assuming a constant wall temperature or a constant heat flux at the wall. In condensers, this wall temperature or heat flux at the wall is in general not known a prior, and the temperature of the cooling fluid (e.g. water) has normally been used as an approximation for the wall temperature. This may be a valid approximation when the mass flow rate of the cooling water is much larger than that of the gas–vapour mixture or when the mass fraction of the water vapour in the gas–vapour mixture is low. However, a better approach is to solve the heat and mass balance of the condensers including the cooling water and the gas–vapour simultaneously. Recently, Li, Saraireh and Thorpe [8] have undertaken the predictions of vapour condensation and heat transfer in the presence of non-condensable gases involving water vapour condensation in gas–vapour mixture flows with water as the cooling fluid. The equations, in combination with many theoretical models for heat and mass transfer for the gas–vapour mixture, were solved numerically and the predictions were found to compare favourably with available experimental results from the literature.

Recently, modelling of water vapour condensation in the presence of non-condensable gases has been conducted using computational fluid dynamics (CFD). The advantages of using CFD include the ability of predicting water vapour condensation in complex geometries and of less assumptions in modelling the mass and heat transfer involved. Revankar and Pollock [9] predicted the laminar film condensation in a vertical tube in the presence of non-condensable gas and the predictions were compared with experimental data. Rao et al. [10] presented the convective condensation of water vapour in the presence of a non-condensable gas of high concentration in laminar flow in a vertical pipe. They predicted the local and average values of the condensation Nusselt number, condensate Reynolds number, gas–liquid interface temperature and pressure drop. Bucci et al. [11] used a commercial and an in-house CFD code to evaluate the heat and mass transfer occurring over a flat plate exposed to an air–vapour stream with uniform bulk stream mass fraction and temperature conditions at the wall. Benelmir, Mokraoui and Souayed [12] conducted a numerical analysis of film-wise condensation in a plate fin-and-tube heat exchanger in the presence of non-condensable gas. Moukalled et al. [13] used CFD to predict and optimize the performance of an air-conditioning equipment. In these CFD simulations of vapour condensation in the presence of non-condensable gas, the condensate film is neglected and the vapour condensation is modelled as a sink term for the mass conservation and species conservation. Mimouni et al. [14] used CFD to model the wall steam condensation using two-phase flow approach and compared the predictions with that using a homogeneous flow approach.

Yuann [15], Panday [16] and Groff et al. [17] solved the governing conservation equations in both the liquid film and the vapour–gas mixture and linked them with interfacial boundary conditions. In Groff et al. [17], the cylindrical coordinate system was transformed such that the interface between the gas mixture and liquid condensate is at a constant *η* = 1 and a set of seven boundary conditions was supplied at the liquid–mixture interface. To solve the conservation equations numerically, the number of grids in both the liquid region and the mixture region were set at the same order of magnitude. Given the large difference in densities between the liquid region and gas mixture region, the thickness of the condensation film is in general three orders of magnitude less than the tube diameter or channel width of the condenser. Using such a large number of grids in the liquid region shows the challenge of this approach in CFD modelling of vapour condensation in condensers.

Laaroussi, Lauriat and Desrayaud [18] studied the effect of variable density for film evaporation on laminar mixed convection in a vertical channel. They have studied the buoyancy effect due to temperature and mass fraction variations using the Boussinesq approximation. They have found that both thermal buoyancy force and solutal buoyancy force need to be considered. They considered only laminar flows and the maximum mass fraction of vapour was up to 50%. In many industry applications, much higher vapour content can be found.

As in those cases of simple theoretical predictions of vapour condensations in the presence of non-condensable gas, all the CFD simulations mentioned above model heat and mass transfer inside the condenser tubes or channels with a constant wall temperature or constant heat flux at the wall. On the other hand, in nearly all the experiments conducted and industrial applications, vapour condensation cannot exist by itself. The condensers are in general cooled by coolant in the cooling jackets. As the results of Li et al. [8] showed, in case of high mass fraction of water vapour inside the condensers, both the condenser wall temperature and heat flux can vary significantly. Also, as a prediction method, the wall temperature and heat flux should be the consequence of predictions rather than input boundary conditions. Saraireh, Thorpe and Li [19] attempted to predict the vapour condensation of a whole plane condenser using ANSYS FLUENT® for low vapour content and encountered many difficulties.

In this paper, we present the results of modelling the water vapour condensation in the presence of non-condensable gas from medium to high vapour content and the heat transfer in the cooling jacket using FLUENT® in vertical cylindrical tubes. Inside the condenser tubes, we model both the gas mixture region and the liquid film. The modelling of the liquid film is undertaken by using the Nusselt approximation rather than solving a set of conservation equations to save computer resources. We also model the buoyancy effect from the variation of temperature and vapour content without using the Boussinesq approximation.

## 2 Governing equations, turbulence modelling and problem formulation

**Fig. 1:**
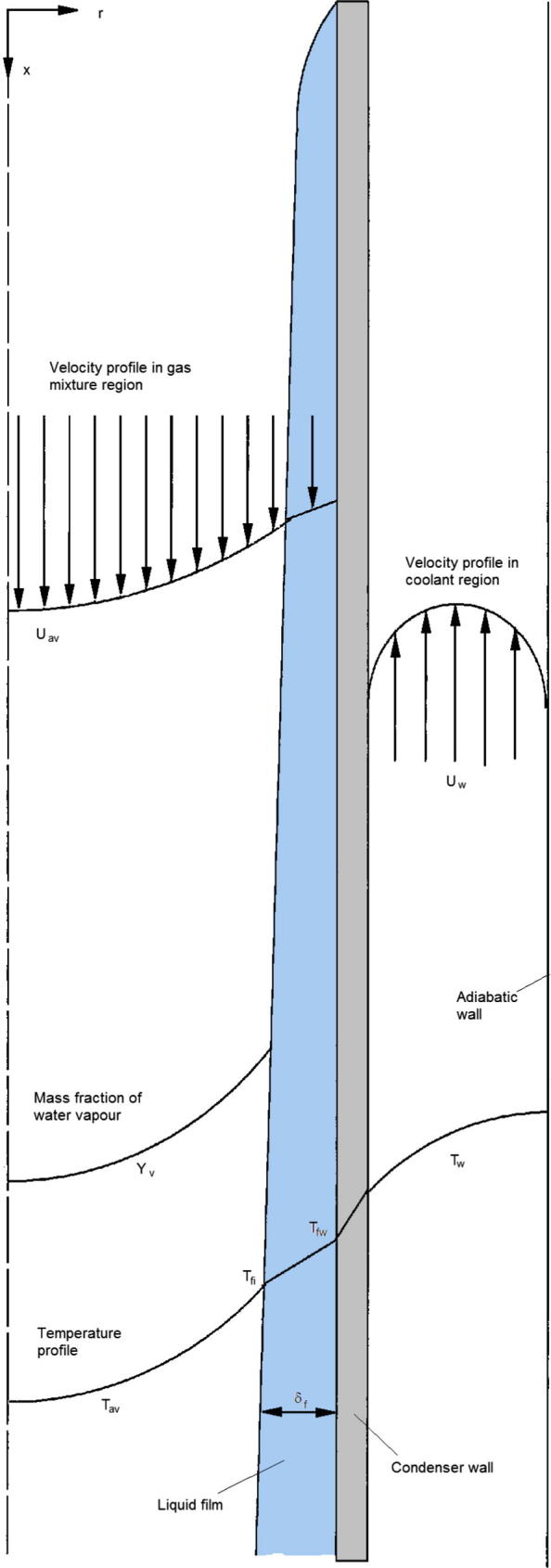
Schematic diagram of the condenser system with defined coordinate systems and quantities.

We consider a vertical condenser tube with an annular cooling channel. Fig. 1 shows a schematic diagram of the condenser system with defined coordinate systems and quantities. Here we assume that the homogeneously mixed air and water vapour mixture enters the condenser tube from the top and the cooling water (coolant) enters the annular channel from the bottom. It is assumed that the condensate forms a thin film on the inner surface of the condenser wall and the film thickness *δ_f_* = 0 at *x* = 0. In addition, we also assume: (1) the flow is statistically steady and axisymmetric; (2) the condensate film is impermeable to non-condensable gas; (3) the thickness of the condensate film is extremely thin and much less than the radius of the condenser tube, *δ_f_* << *r_i_*; (4) the air and water vapour–gas mixture is an ideal gas and its thermodynamic properties vary with temperature; (5) condensation occurs only at the interface between the liquid film and the gas mixture; and (6) the cooling channel is surrounded by an adiabatic wall.

### 2.1 Conservation equations

The conservation of mass or continuity equation can be written as:

(1) $$\frac{\partial }{\partial x}(\rho U)+\frac{\partial }{\partial r}(\rho V)+\frac{\rho V}{r}=S_{m}$$

where *S_m_* is the source terms of total mass. The equations for momentum conservation are

(2) $$\begin{array}{cc} & \frac{\partial }{\partial x}(\rho \mathrm{UU})+\frac{1}{r}\frac{\partial }{\partial r}(r\rho \mathrm{UV})\\ & \;=-\frac{\partial P}{\partial x}+\frac{\partial }{\partial x}\left[\mu_{\mathrm{eff}}\left(2\frac{\partial U}{\partial x}-\frac{2}{3}(\nabla \cdot \overset{\to }{u})\right]\right)\\ & \;+\frac{1}{r}\frac{\partial }{\partial r}\left[r\mu_{\mathrm{eff}}\left(\frac{\partial U}{\partial r}+\frac{\partial V}{\partial x}\right)\right]+\rho g+S_{U}\\ & \frac{\partial }{\partial x}(\rho \mathrm{UV})+\frac{1}{r}\frac{\partial }{\partial r}(r\rho \mathrm{VV})\\ & \;=-\frac{\partial P}{\partial r}+\frac{\partial }{\partial x}\left[\mu_{\mathrm{eff}}\left(\frac{\partial U}{\partial r}+\frac{\partial V}{\partial x}\right)\right]\\ & \;+\frac{1}{r}\frac{\partial }{\partial r}\left[r\mu_{\mathrm{eff}}\left(2\frac{\partial V}{\partial r}-\frac{2}{3}(\nabla \cdot \overset{\to }{u})\right)\right]-2\mu_{\mathrm{eff}}\frac{V}{r^{2}}\\ & \;+\frac{2}{3}\frac{\mu_{\mathrm{eff}}}{r}(\nabla \cdot \overset{\to }{u})+S_{V} \end{array}$$

Here *S_U_* and *S_V_* are the source terms for momentum in *x* and *r* directions, respectively, and

$$\nabla .\overset{\to }{u}=\frac{\partial U}{\partial x}+\frac{\partial V}{\partial r}+\frac{V}{r}$$

The conservation of energy in statistically steady cylindrical coordinate systems are

(3) $$\frac{\partial }{\partial x}(U(\rho E+P))+\frac{1}{r}\frac{\partial }{\partial r}(\mathrm{rV}(\rho E+P))=\frac{\partial }{\partial x}\left(\lambda_{\mathrm{eff}}\frac{\partial T}{\partial x}-\sum h_{i}J_{\mathrm{ix}}+{\left({\overset{¯}{\overset{¯}{\tau }}}_{\mathrm{eff}}.\overset{\to }{v}\right)}_{x}\right)+\frac{1}{r}\frac{\partial }{\partial r}\left(r\lambda_{\mathrm{eff}}\frac{\partial T}{\partial r}-\sum h_{i}J_{\mathrm{ir}}+{\left({\overset{¯}{\overset{¯}{\tau }}}_{\mathrm{eff}}\cdot \overset{\to }{v}\right)}_{r}\right)+S_{h}$$

where ${\overset{¯}{\overset{¯}{\tau }}}_{\mathrm{eff}}$ is the turbulent shear stress tensor (2D), $\overset{\to }{v}$ is the velocity vector and *S_h_* is the source term for energy.

In the above equation

$$E=h-\frac{P}{\rho }+\frac{\left(U^{2}+V^{2}\right)}{2}$$

where *h* is the sensible enthalpy and for compressible flows it is defined as:

(4) $$h=\underset{i}{\sum }Y_{i}h_{i}$$

and $h_{i}=\int_{T_{\mathrm{ref}}}^{T}C_{p\text{,}i}\mathrm{dT}$ with the reference temperature *T_ref_* = 298.15 K is used.

For the annular channel flow of cooling water, only water liquid is flowing. The fluid can be considered as incompressible and

$$E=h+\frac{\left(U^{2}+V^{2}\right)}{2}$$

For the gas mixture flow inside the tube condenser, the mass fraction of the water vapour satisfies the following equation

(5) $$\frac{\partial }{\partial x}\left(\rho {\mathrm{UY}}_{v}\right)+\frac{1}{r}\frac{\partial }{\partial r}\left(r\rho {\mathrm{VY}}_{v}\right)=-\left(\frac{\partial J_{v\text{,}x}}{\partial x}+\frac{1}{r}\frac{\partial ({\mathrm{rJ}}_{v\text{,}r})}{\partial r}\right)+S_{v}$$

$$\begin{array}{cc} & J_{v\text{,}x}=-\left(\rho D_{v\text{,}m}+\frac{\mu_{\mathrm{eff}}}{{\mathrm{Sc}}_{t}}\right)\frac{\partial Y_{v}}{\partial x}-\frac{D_{T\text{,}v}}{T}\frac{\partial T}{\partial x}\\ & J_{v\text{,}r}=-\left(\rho D_{\mathrm{vm}}+\frac{\mu_{\mathrm{eff}}}{{\mathrm{Sc}}_{t}}\right)\frac{\partial Y_{v}}{\partial r}-\frac{D_{T\text{,}v}}{T}\frac{\partial T}{\partial r} \end{array}$$

where *S_v_* is the source (sink) term for the water vapour, *Sc_t_* is the turbulent Schmidt number, *D_v_*_,_*_m_* is the mass diffusivity of water vapour and air, and *D_T_*_,_*_v_* is the thermal diffusivity. The conservation equations are completed by the ideal gas law for the gas mixture

$$\begin{array}{cc} & \rho =\frac{\mathrm{PM}}{\mathrm{RT}}\\ & Y_{a}+Y_{v}=1 \end{array}$$

and the molar mass of the mixture *M* is calculated from

$$\frac{1}{M}=\left(\frac{Y_{v}}{M_{v}}+\frac{Y_{a}}{M_{a}}\right)$$

### 2.2 Turbulence modelling

The realizable *k*–*ɛ* model [20] was used to model turbulence in the present simulation. The governing equations for the turbulent kinetic energy *k* and the dissipation rate *ɛ* are

(6) $$\begin{array}{cc} & \frac{\partial }{\partial x}(\rho \mathrm{Uk})+\frac{1}{r}\frac{\partial }{\partial r}(r\rho \mathrm{Vk})\\ & \;=\frac{\partial }{\partial x}\left[\left(\mu +\frac{\mu_{t}}{\sigma_{k}}\right)\frac{\partial k}{\partial x}\right]+\frac{1}{r}\frac{\partial }{\partial r}\left[r\left(\mu +\frac{\mu_{t}}{\sigma_{k}}\right)\frac{\partial k}{\partial r}\right]\\ & \;+G_{k}+G_{b}-\rho \varepsilon +S_{k}\\ & \frac{\partial }{\partial x}(\rho U\varepsilon )+\frac{1}{r}\frac{\partial }{\partial r}(r\rho V\varepsilon )\\ & \;=\frac{\partial }{\partial x}\left[\left(\mu +\frac{\mu_{t}}{\sigma_{\varepsilon }}\right)\frac{\partial \varepsilon }{\partial x}\right]+\frac{1}{r}\frac{\partial }{\partial r}\left[r\left(\mu +\frac{\mu_{t}}{\sigma_{\varepsilon }}\right)\frac{\partial \varepsilon }{\partial r}\right]\\ & \;+\rho C_{1}S\varepsilon -\rho C_{2}\frac{\varepsilon^{2}}{k+\sqrt{\nu \varepsilon }}+C_{1\varepsilon }\frac{\varepsilon }{k}C_{3\varepsilon }G_{b}+S_{\varepsilon } \end{array}$$

In these equations, *G_k_* represents the generation of turbulent kinetic energy due to the mean velocity gradients and *G_b_* is the generation of turbulence kinetic energy due to buoyancy. The contribution of the fluctuating dilatation in compressible turbulence to the overall dissipation rate has been neglected. *S_k_* and *S_ɛ_* are user-defined source terms. In the above equations,

$$C_{1}=\max\left[0.43\text{,}\;\frac{\eta }{\eta +5}\right]\text{,}\;\eta =S\frac{\varepsilon }{k}\text{,}\;S=\sqrt{2S_{\mathrm{ij}}S_{\mathrm{ij}}}$$

In the turbulence modelling,

$$\begin{array}{cc} & \mu_{t}=\rho C_{\mu }\frac{k^{2}}{\varepsilon }\\ & \mu_{\mathrm{eff}}=\mu +\mu_{t} \end{array}$$

The special feature of realizable *k*–*ε* model is that *C_μ_* is not a constant, rather it is calculated as

$$C_{\mu }=\frac{1}{A_{0}+A_{s}\frac{{\mathrm{kU}}^{*}}{\varepsilon }}\text{,}\;U^{*}=\sqrt{S_{\mathrm{ij}}S_{\mathrm{ij}}+{\overset{⌢}{\Omega }}_{\mathrm{ij}}{\overset{⌢}{\Omega }}_{\mathrm{ij}}}\text{,}\;{\overset{⌢}{\Omega }}_{\mathrm{ij}}=\Omega_{\mathrm{ij}}-2\varepsilon_{\mathrm{ijk}}\omega_{k}\;\Omega_{\mathrm{ij}}={\overset{¯}{\Omega }}_{\mathrm{ij}}-\varepsilon_{\mathrm{ijk}}\omega_{k}$$

Here ${\overset{¯}{\Omega }}_{\mathrm{ij}}$ is the mean rate of rotation tensor viewed in a moving frame with angular velocity *ω_k_* and

$$\begin{array}{cc} & A_{0}=4.04\text{,}\;A_{s}=\sqrt{6}\cos\;\varphi \\ & \varphi =\frac{1}{3}\cos^{-1}(\sqrt{6}W)\text{,}\;W=\frac{S_{\mathrm{ij}}S_{\mathrm{jk}}S_{\mathrm{ki}}}{{\overset{˜}{S}}^{3}}\text{,}\;\overset{˜}{S}=\sqrt{S_{\mathrm{ij}}S_{\mathrm{ij}}}\text{,}\;S_{\mathrm{ij}}=\frac{1}{2}\left(\frac{\partial u_{i}}{\partial x_{j}}+\frac{\partial u_{j}}{\partial x_{i}}\right) \end{array}$$

The model constants are

$$C_{1\varepsilon }=1.44\text{,}\;C_{2}=1.9\text{,}\;\sigma_{k}=1.0\text{,}\;\sigma_{\varepsilon }=1.2$$

### 2.3 Source terms

In simulating the flows in the condenser system as that shown in Fig. 1, various source terms exist at the interface between the gas mixture and condensate film. These need to be specified during the CFD simulation.

#### 2.3.1 Source term for mass

The source terms for the total mass and the water vapour are due to the condensation of water vapour at the interface between the gas mixture and the condensate film and is calculated using

(7) $$S_{m}=S_{v}={\left(\frac{\rho D_{v\text{,}m}}{1-Y_{v}}\frac{\partial Y_{v}}{\partial r}\right|}_{r=r_{i}}\frac{\delta A}{\delta V}$$

where *δA* is the surface area and *δV* is the cell volume next to the interface. Here we assume that the gas mixture is extended to the inner surface of the condenser tube and the thickness of the condensate film is negligible. The mass fraction of the water vapour at the interface is calculated by assuming that the gas mixture–liquid film is in thermodynamic equilibrium and is related to the partial pressure of water vapour at saturation condition by

(8) $${\left(Y_{v}\right|}_{r=r_{i}}=\frac{M_{v}}{M_{a}}\frac{P_{\mathrm{sat}}\left(T_{\mathrm{fi}}\right)}{P-\left(1-\frac{M_{v}}{M_{a}}\right)P_{\mathrm{sat}}(T_{\mathrm{fi}})}$$

where *P* is the local total pressure, *P_sat_*(*T_fi_*) is the saturation vapour pressure at the interface temperature and is calculated as [10]

(9) $$P_{\mathrm{sat}}(T)=(\exp(77.3448+0.005713T-7235/T)/T^{8.2}$$

#### 2.3.2 Source term for momentum

Because of the large variation of temperature and mass fraction of water vapour, the density of the gas mixture will vary significantly within the condenser tube. The first consequence of this is that the flow inside the condenser tube cannot be assumed to be incompressible which requires constant density. The large variation of density across the flow field also results in large buoyancy forces. As discussed by Laaroussi et al. [18], both thermal and solutal buoyancy forces exist in the condensation and evaporation of gas mixture with high vapour content. In Laaroussi et al. [18], the buoyancy forces were modelled as source terms in the *x* direction momentum equation using Boussinesq approximation, even though this approximation is valid only for small temperature differences, and the thermo-physical properties of the mixture were evaluated at reference temperature and mass fraction (vary with the vertical channel) given by the 1/3 law from the expression given by Fujii et al. [21].

When using the Boussinesq approximation for the problem involving concentration variation as well as temperature variation, the density of the mixture is approximated by a double Taylor expansion (Bird, Stewart and Lightfoot [22]) as

(10) $$\begin{array}{cc} & \rho (T\text{,}Y_{v})=\overset{¯}{\rho }+{\left(\frac{\partial \rho }{\partial T}\right|}_{\overset{¯}{T}\text{,}{\overset{¯}{Y}}_{v}}(T-\overset{¯}{T})+{\left(\frac{\partial \rho }{\partial Y_{v}}\right|}_{\overset{¯}{T}\text{,}{\overset{¯}{Y}}_{v}}\left(Y_{v}-{\overset{¯}{Y}}_{v}\right)+\cdots \\ & \;\approx \overset{¯}{\rho }-\overset{¯}{\rho }\overset{¯}{\beta }(T-\overset{¯}{T})-\overset{¯}{\rho }\overset{¯}{\zeta }(Y_{v}-{\overset{¯}{Y}}_{v})\\ & \overset{¯}{\beta }={\left(-\frac{1}{\overset{¯}{\rho }}\frac{\partial \rho }{\partial T}\right|}_{\overset{¯}{T}\text{,}{\overset{¯}{Y}}_{v}}\\ & \overset{¯}{\zeta }={\left(-\frac{1}{\overset{¯}{\rho }}\frac{\partial \rho }{\partial Y_{v}}\right|}_{\overset{¯}{T}\text{,}{\overset{¯}{Y}}_{v}} \end{array}$$

Under this approximation, the momentum equation can be written as (Bird et al. [22])

(11) $$\frac{D(\rho V)}{\mathrm{Dt}}=(-\nabla P+\overset{¯}{\rho }g)-\overset{¯}{\rho }g\overset{¯}{\beta }(T-\overset{¯}{T})-\overset{¯}{\rho }g\overset{¯}{\zeta }(Y_{v}-{\overset{¯}{Y}}_{v})+\cdots$$

In the above equation, the pressure *P* can be redefined to include the hydrostatic force due to density variation (Batchelor [23]), the buoyancy forces are due to the linear variations of the temperature and mass fraction of the water vapour. Fig. 1 shows that for the current problem, both the average temperature and the average mass fraction of the water vapour along the condenser tube decrease as the gas mixture flow from the top of the tube to the bottom of the tube. This results in an increase in average density of the gas mixture along the condenser tube. Across the condenser tube, the temperature and mass fraction of the water vapour also decrease from the tube centre to the condensate film. This results in, on average, a density increase of the gas mixture towards the condensate film. Instead of using the Boussinesq approximation of expanding the density variation as that due to small variation of temperature and mass fraction of water vapour, we write the *x* direction momentum equation as

(12) $$\frac{D(\rho U)}{\mathrm{Dt}}=\left(-\frac{\partial P}{\partial x}+\rho_{0}g\right)+(\rho -\rho_{0})g+\cdots$$

Here *ρ*_0_ is the density of the gas mixture at the centre of the condenser tube and increase with *x*. The above buoyancy force was applied in the gas mixture region and no linear approximation is involved.

At the interface between the gas mixture and the condensate film, as water vapour condenses into water liquid, it will cause a loss of momentum in the gas mixture and this can be modelled as

(13) $$\begin{array}{cc} & S_{U}={\mathrm{US}}_{m}\\ & S_{V}={\mathrm{VS}}_{m} \end{array}$$

Together with the buoyancy force in the *x* direction, these are added to the momentum equation (2) in the CFD simulation of the gas mixture region.

#### 2.3.3 Source term for energy

Similarly, the water vapour condensation also causes the removal of energy from the mixture region. This can be calculated as

(14) $$S_{h}=S_{m}h_{v}$$

This is different from that commonly used

$$S_{h}=S_{m}\left(h_{v}-h_{a}\right)$$

Since the condensation occurs at the interface between the gas mixture and condensate film where only a sink term exists for the water vapour and there is no source term for the non-condensable gas.

### 2.4 Boundary conditions

For the cooling annular channel, the outside tube is assumed to be adiabatic and the mass flow rate and the temperature at its inlet are specified as

$$\overset{˙}{m}={\overset{˙}{m}}_{c}\text{,}\;T_{w}=T_{c}\;\mathrm{at}\;x=L$$

A pressure outlet boundary condition is specified for the coolant at the exit.

For the gas mixture region, the total mass flow rate, the temperature and the mass fraction of water vapour are specified at the inlet

$${\overset{˙}{m}}_{\mathrm{av}}={\overset{˙}{m}}_{\mathrm{av}\text{,}0}\text{,}\;T_{\mathrm{av}}=T_{\mathrm{av}\text{,}0}\text{,}\;Y_{v}=Y_{v\text{,}0}\;\mathrm{at}\;x=0$$

The flow is assumed to be normal to the inlets.

As in Li et al. [8], the condensate film is modelled as a very thin layer. Instead of solving the conservation equations in the condensate film, we conduct the CFD simulation only for the gas mixture region in the condenser tube and the condensate film is considered as providing the necessary boundary conditions. By considering the balance between the weights of the fluid elements, the buoyancy force and the viscous shear force, the velocity gradient of the condensate in the film can be written as (Li et al. [8])

(14) $$\frac{\mathrm{du}}{\mathrm{dy}}=\frac{\left(\rho_{l}-\rho \right)}{\mu_{l}}\left(\delta_{f}-y\right)+\frac{\tau_{g}}{\mu_{l}}$$

where *y* is the distance from the condenser inner surface, *ρ_l_* is the density of the condensate, *μ_f_* is the dynamic viscosity of the condensate and *τ_g_* is the shear stress at the interface between the air–vapour mixture and the condensate film. By integration, the velocity distribution in the condensate film can then be derived as

$$u=\frac{1}{2}\frac{\left(\rho_{l}-\rho \right)g}{\mu_{l}}\left[\delta_{f}^{2}-{\left(\delta_{f}-y\right)}^{2}\right]+\frac{\tau_{g}}{\mu_{l}}y$$

This shows that the velocity at the surface of the condensate film is

$$u=\frac{1}{2}\frac{\left(\rho_{l}-\rho \right)g}{\mu_{l}}\delta_{f}^{2}+\frac{\tau_{g}}{\mu_{l}}\delta_{f}$$

Here we have assumed that the velocity of the condensate is zero at the inner surface of the condenser wall (*r_i_*). We assume that at the interface of the gas mixture and the condensate film, the velocity of the gas mixture is the same as that of the liquid film (non-slip). Thus

(15) $$U=\frac{1}{2}\frac{\left(\rho_{l}-\rho \right)g}{\mu_{l}}\delta_{f}^{2}+\frac{\tau_{g}}{\mu_{l}}\delta_{f}\text{,}\;V=0\;\mathrm{at}\;r=r_{i}$$

The reason for specifying *V* = 0 at *r = r_i_* is that the gas mixture cannot flow into the condensate film. During CFD simulations, the *τ_g_* was obtained from the wall shear stress of the gas mixture. The mass flow rate of the condensate film can be calculated from

(16) $${\overset{˙}{m}}_{f}=2\pi \rho_{l}\left[\frac{\left(\rho_{l}-\rho \right)g}{\mu_{l}}\left(\frac{2}{3}r_{i}\delta_{f}^{3}-\frac{5}{12}\delta_{f}^{4}\right)+\frac{\tau_{g}}{\mu_{l}}\left(\frac{1}{2}r_{i}\delta_{f}^{2}-\frac{1}{3}\delta_{f}^{3}\right)\right]$$

At *x* = 0, *δ_f_* = 0. The mass flow rate of the condensate film at each *x* location is related to the vapour condensation at the interface between the gas mixture and the film and

(17) $${\overset{˙}{m}}_{f}=2\pi r_{i}\int_{0}^{x}{\left(\frac{\rho D_{v\text{,}m}}{1-Y_{v}}\frac{\partial Y_{v}}{\partial r}\right|}_{r=r_{i}}\mathrm{dx}$$

By combining the above two equations, the thickness of the condensate film *δ_f_* can be determined at each *x* location. This film thickness is used to determine the temperature of the condensate at the film surface

(18) $$\begin{array}{cc} & q^{\prime \prime }=\frac{\lambda_{f}\left(T_{f\text{,}i}-T_{f\text{,}w}\right)}{\left(r_{i}-\delta_{f}\right)\ln\left[r_{i}/\left(r_{i}-\delta_{f}\right))\right]}\\ & T_{f\text{,}i}=T_{f\text{,}w}\;\mathrm{if}\;\delta_{f}=0 \end{array}$$

where *λ_f_* is the thermal conductivity of the condensate, *T_f,w_* is the temperature of the inner surface of the condenser tube and the heat flux $q^{\prime \prime }$ at the interface is calculated as

(19) $$q^{\prime \prime }=-\lambda_{m}\frac{\partial T}{\partial r}-\frac{\rho D_{v\text{,}m}h_{l\text{,}v}}{\left(1-Y_{v}\right)}\frac{\partial Y_{v}}{\partial r}$$

Here *λ_m_* is the thermal conductivity of the gas mixture at the interface and *h_l,v_* is the latent heat released by the water vapour during condensation.

As condensation occurs along the inner surface of the condenser tube, the condensate film changes from smooth laminar flow to rough turbulent flow. As in Li et al. [8], we assume that the interface between the gas mixture and the condensate film is rough. It is assumed that the roughness height is half the film thickness *δ_f_*.

A pressure outlet boundary condition is specified at the exit of the condenser tube. In case of reverse flows at the exit of the condenser (*U* < 0), the backflow boundary conditions for temperature and mass fraction of water vapour are specified using

(20) $$T=(\underset{U>0}{\sum }{\overset{˙}{m}}_{\mathrm{av}\text{,}j}T_{j}/\underset{U>0}{\sum }{\overset{˙}{m}}_{\mathrm{av}\text{,}j}\text{,}\;Y_{v}=(\underset{U>0}{\sum }{\overset{˙}{m}}_{\mathrm{av}\text{,}j}Y_{\mathrm{vj}}/\underset{U>0}{\sum }{\overset{˙}{m}}_{\mathrm{av}\text{,}j}\text{,}$$

This assumes that the backflow is from the nearby forward flow and temperature and mass fraction of the water vapour for the backflow is assumed to be the average temperature and mass fraction of water vapour of the forward flow at the exit of the condenser tube. During CFD simulations, at the intermediate iteration steps, the whole exit can be back flow. In this case, *T* = 300 K and *Y_v_* = 0 are used at the boundary conditions for the backflow.

## 3 Solution procedures

The simulations of heat and mass transfer in the condenser as shown in Fig. 1 were carried out using commercially available FLUENT®, now part of ANSYS®. As pointed out in Saraireh et al. [19] in using FLUENT® for predicting the heat and mass transfer in both the air–vapour mixture channel and the cooling water channel simultaneously, many challenges are encountered. As stated in the FLUENT® user’s guide, in modelling heat transfer in two separated fluid regions involving multispecies, only a single mixture material for the entire domain can be used [24]. Because of this, the two flows in the present situation including the air–vapour mixture and the cooling water cannot be simulated simultaneously using FLUENT® since the flow in one channel is a mixture of air and water vapour and in the other channel the flow is water liquid. FLUENT20 can model two flows separated by a solid wall only if one flow is water liquid, say, and the other is a single species such as air or water vapour alone. Saraireh et al. [19] have attempted the simulation using various methods and suggested that the flows in the condenser and the cooling channel can be separated into two and the simulations are carried them out asynchronously. The heat and mass transfer is analysed in the gas mixture in the tube condenser and the heat transfer is analysed for both the cooling water in the annular channel and the stainless steel condenser tube wall. The two simulations are coupled at the inner surface of the stainless steel condenser tube. The flow in the gas mixture condenser was simulated first. A guessed wall temperature from a pre-written file (this file includes the temperature at the condensing surface at each grid centre) at the inner surface of the condenser tube was read first and the simulation was carried out until convergence was achieved. A separate file was written for the heat flux ${\overset{˙}{Q}}^{\prime \prime }$ at the condenser surface as an output of this simulation. Heat transfer in the cooling water channel and the stainless tube was then simulated using the heat flux file written previously as the input boundary conditions. The simulation was again carried out to convergence and a file for temperature *T_f,w_* at the inner surface of the condensing tube was written as an output of this second simulation. A journal file was written to run the two simulations alternatively many times to achieve convergence for flows in both the gas mixture and cooling water regions.

In simulating the flow in the condenser tube, a gas mixture of air and water vapour was introduced at the inlet with a given mass flow rate, temperature and mass fraction of the water vapour. All the thermal properties of the air and water vapour were assumed to be functions of temperature and were calculated in user-defined functions (UDF). The source terms mentioned early were also calculated using UDF and hooked to their corresponding conservation equations. Because the buoyancy force was calculated as a source term for momentum equation (2), no Boussinesq approximation was used for simulating the gas mixture flows.

In simulating the flow in the cooling channel, water was introduced at the inlet (from the bottom as shown in Fig. 1) at the prescribed mass flow rate and temperature. Boussinesq approximation was used to model the buoyancy effects as recommended by Li et al. [8], because large difference in temperature between the condenser wall and the bulk temperature of the coolant can exist, buoyancy effects cannot generally be neglected in the cooling channel. In the cooling channel, the density variation of the water liquid is considered small and Boussinesq approximation can be expected to work adequately. The thermal properties of the cooling water were allowed to vary with temperature and these were calculated using UDF for the cooling channel.

To use the wall temperature profile (written in a file in the simulations for cooling channel and stainless condenser tube) in the simulation of flows in the air–steam mixture channel and to use the heat flux profile (written in a file in the simulations for mixture flow) in the simulation of cooling water and stainless condenser tube, the grids on the surface of the condensing wall common to both simulations need to be matched. Also, in general, FLUENT® performs CFD simulations starting from the inlet of the fluid domain and the positions at the condensing surface in the two simulations needs to be carefully matched.

To avoid divergence, the following measures were taken for the simulation of gas mixture region: the source term for mass has been under-relaxed as

(21) $$S_{m}^{n}=(1-\alpha )S_{m}^{n-1}+\alpha \left[{\left(\frac{\rho D_{v\text{,}m}}{1-Y_{v}}\frac{\partial Y_{v}}{\partial r}\right|}_{r=r_{i}}\frac{\delta A}{\delta V}\right]$$

where the second term on the r.h.s. of the equation is the source term in Eq. (7) times an under-relaxation factor *α*, *n* is the *n*th iteration and *α* = 0.05 is used.

In solving the present problem using CFD, a third order MUSCL discretization scheme was used for all the conservation equations. The pressure–velocity coupling was solved using a coupled scheme and the pressure was calculated with a body-force weighted scheme. The solver used was pressure based and the flow is assumed to be steady and axisymmetric. User-defined functions (UDFs) were written for all the source terms, boundary conditions as given in Section 2.4 and the properties of the fluids and were called at each iteration.

## 4 Results and discussion

The CFD simulations were conducted using the condenser of the same dimension as that used in the experimental work of Kuhn [6]. In Li et al. [8], the experimental results of Siddique [5], Tanrikut and Yesin [25] and Kuhn [6] were compared with model predictions. It was found that the measured centreline temperature of the condenser from Tanrikut and Yesin [25] was close to the wet bulb temperature rather than dry bulb temperature. Sddique [5] conducted the experiments by using turbulence promoters for the coolant channel in order to represent the bulk temperature of the coolant channel using the measured middle channel temperature. In CFD simulations, it is difficult to specify the turbulent intensity at the inlet of the coolant channel since no information is given in the experimental results of Sddique [5]. Because of these, we compare with the experimental results of Kuhn [6] here only. The test sections of the experiments in Kuhn [6] were circular, vertical and metallic tubes, surrounded by annular jackets through which a liquid coolant (water liquid) flowed. The gas–vapour mixture flowed downward in the tube while the coolant in the jacket flowed upward. The condenser tube in the experiments of Kuhn [6] was stainless and 2.418 m long but the experimental results were presented only for distance up to 1.48 m from the inlet of the air–vapour mixture. The experiments of Kuhn [6] were conducted for pure steam, steam–air mixtures and steam–helium mixtures. Kuhn [6] also investigated the effect of turbulent condensate films on the heat transfer by using suitable film distributors. In this paper, we compare the experimental results from Kuhn [6] only for the steam–air mixture experiments with no arbitrarily introduced condensate film. In Kuhn [6], the coolant bulk temperature was not directly measured, rather it was estimated by measuring the temperatures at the inner and outer walls of the annulus and by calculating the temperature difference ratio (defined as a shape factor F) numerically. Table 1 shows the selected experimental conditions for some of the experiments from Kuhn [6]. Kuhn [6] repeated some of the experiments to confirm the results and the inlet mass fraction of the water vapour for the runs given in Table 1 varies from 0.66 to 0.98.

In the experiments of Kuhn [6], the local heat flux was estimated using

(22) $$q^{\prime \prime }=\frac{{\overset{˙}{m}}_{c}C_{p}}{\pi d}\frac{{\mathrm{dT}}_{c}}{\mathrm{dL}}(x)$$

where *T_c_* is the estimated bulk temperature of the coolant and the slope *dT_c_/dL* was estimated from a least square curve fit as a function of condenser length. The condensation rates were then estimated using

(23) $${\overset{˙}{m}}_{\mathrm{cond}}=\frac{\overset{˙}{Q}}{h_{\mathrm{fg}}}$$

Here ${\overset{˙}{m}}_{\mathrm{cond}}$ is the total condensation rate (or the total condensation rate as collected), $\overset{˙}{Q}$ is the total heat transfer rate across the condenser wall, and *h_fg_* is the latent heat of condensation and was calculated using the average temperature of the condenser wall. In using Eq. (23) to calculate the total condensation rate, the contribution of the sensible heat transfer in $\overset{˙}{Q}$ was neglected. It needs to be pointed that the heat flux and the condensation rates given in Kuhn [6] are derived results. The directly measured results are the centreline temperature of the condenser tube, the condenser tube wall temperature and the temperature of the adiabatic wall.

In the present CFD simulation, the condenser tube, the condenser wall and the coolant jacket are discretised into 800 uniform grids in the axial direction. In the radial direction, the condenser tube is discretised into 50 non-uniform grids with a bias ratio of 5 with the smallest grid near the inner surface of the condenser wall, the condenser wall has 4 uniform grids and the coolant jacket has 20 uniform grids. Tests were conducted using a number of grids in both the radial and axial directions and the results show that the resolutions used are adequate and the results for total condensation rate, wall temperature and heat flux are grid independent.

**Fig. 2:**
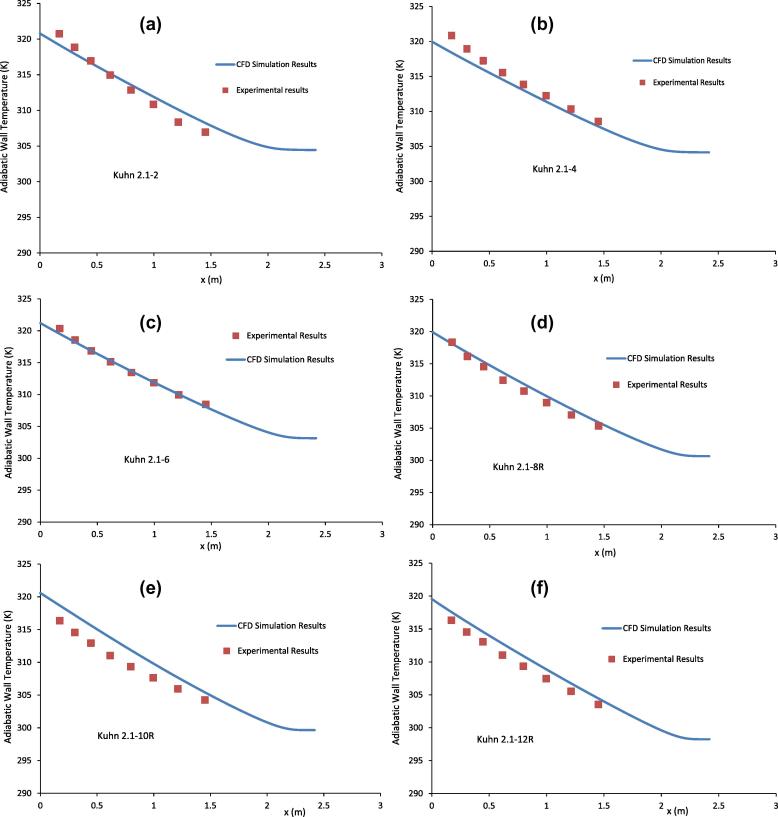
Comparison of adiabatic wall temperature between the CFD simulation results and the experimental results of Kuhn [6] for the runs listed in Table 1.

Fig. 2(a-f) show the comparison of adiabatic wall temperature between the CFD simulation results and the experimental results of Kuhn [6] for the runs listed in Table 1.

Fig. 2(a–f) show that the CFD simulation results for the adiabatic wall temperature in general agree well with the experimental results of Kuhn [6] for *x* < 1.5 m. The maximum difference between the CFD simulation results and the experimental data is about 2 °C. The CFD simulation results show that near the inlet of the coolant jacket (*x* = 2.418 m), the adiabatic wall temperature is almost constant for some distance from the inlet. A close examination of the CFD results shows that the length of the coolant jacket where the adiabatic wall temperature is constant and close to that of the coolant inlet temperature depends on the mass flow rate of the coolant. Using the data from Table 1 and the results in Fig. 2(a-f), it can be seen that the higher the mass flow rate of the coolant, the longer the region where the adiabatic wall temperature will remain constant. This is because the higher is the mass flow rate, the higher the velocity of the coolant in the jacket. This high velocity will carry the coolant faster than the heat transfer from the condenser tube to the adiabatic wall near the inlet of the coolant and thus results in a longer distance for the temperature of the adiabatic wall to change.

**Fig. 3:**
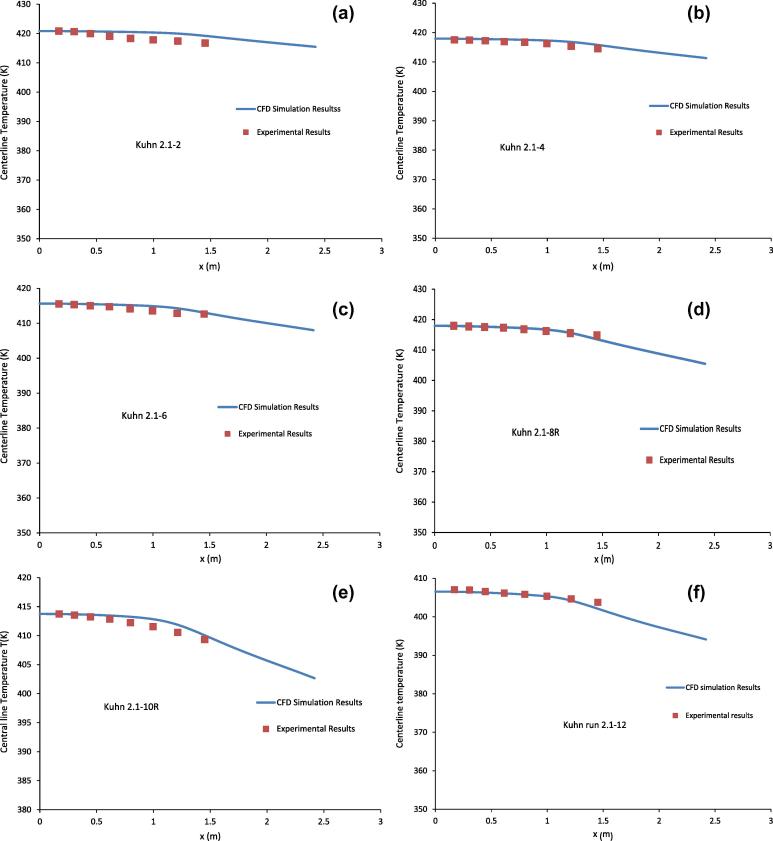
Comparison of centreline temperature between the CFD simulation results and the experimental results of Kuhn [6] for the runs listed in Table 1.

Fig. 3(a-f) show the comparison of the centreline temperature of the condenser tube between the CFD simulation results and the experimental results of Kuhn [6] for the runs listed in Table 1. It can be seen from Fig. 3(a-f), the CFD simulation results in general agree very well with the experimental results. All the CFD simulation results show that the centreline temperature of the condenser tube is almost constant for *x* < 1.0 m. Fig. 3(a-f) show that, after the initial near constant value, the centreline temperature decreases faster with increasing *x*. By using the data as given in Table 1 and the results shown in Fig. 3(a-f), it can be concluded that the rate of temperature decrease depends on the inlet mass fraction of the water vapour. The lower is the inlet mass fraction of water vapour, the faster the centreline temperature decreases as can be seen from Fig. 3(d-f) where the centreline temperature decreases faster at x > 1.0 m than that in Fig. 3(a-c). This is because at x < 1.0 m in Fig. 3(d-f), the heat transfer is dominated by vapour condensation (latent heat) which involves little temperature change. For x > 1.0 m, vapour content is relatively low, the contribution to heat transfer from the sensible heat will be increasing, which will involve large temperature decrease.

**Fig. 4:**
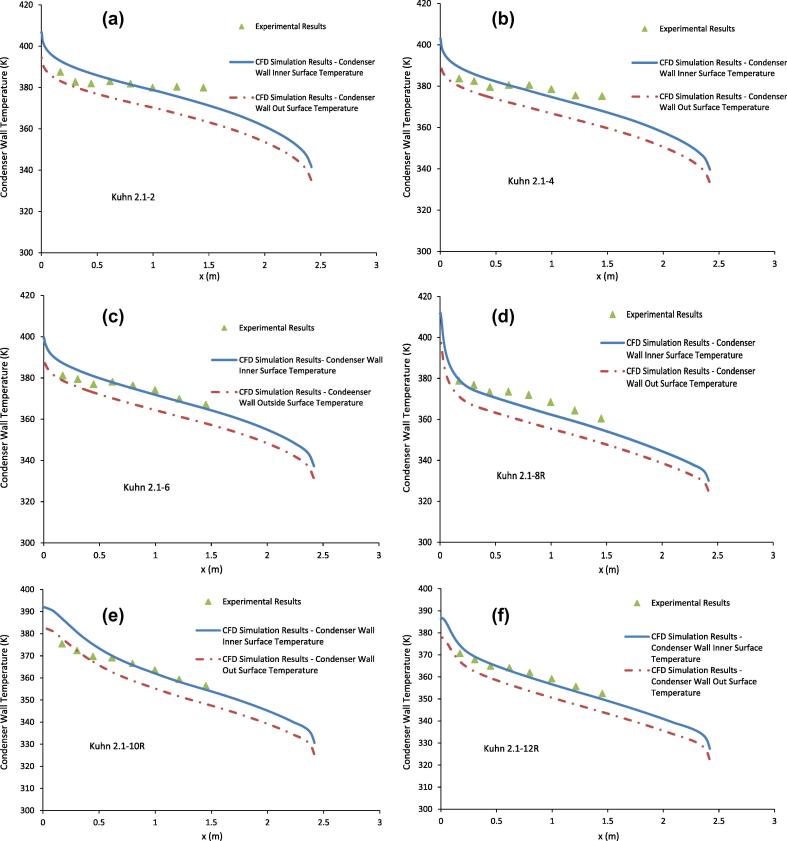
Comparison of condenser wall temperature between the CFD simulation results and the experimental results of Kuhn [6] for the runs listed in Table 1.

Fig. 4(a-f) show comparisons of condenser wall temperature between the CFD simulation results and the experimental results of Kuhn [6] for the runs listed in Table 1. In Kuhn [6], the condenser wall temperature was measured by using J-type thermocouples with 0.508-mm diameter soldered into longitudinal grooves of 0.7-mm wide, 0.58-mm deep and 12.7-mm long machined close to the outer surface of the condenser tube. Because of this, the measured wall temperature should be between the temperatures of the outer and inner surfaces of the condenser wall. Since the wall thickness of the condenser tube is 1.65 mm, it is expected that the measured condenser wall temperature should be closer to that of the outer surface than that at the inner surface. In Fig. 4(a-f), the temperatures at the condenser inner and outer surfaces are both presented. The results in Fig. 4(a-f) show that the temperatures of the condenser tube surfaces predicted from the CFD simulations in general agree reasonably well with the measured tube temperatures. Fig. 4(a-f) show that the wall temperature measured by Kuhn [6] is in general higher than the wall temperature of the inner surface as given by the CFD simulation.

As pointed out by Li et al. [8], the wall temperature of the condenser tube is not constant and it varies significantly over the length of the condenser tube. The results from Fig. 4(a-f) show that the temperature of the condenser wall changes from that close to the inlet temperature of the coolant to that close to the inlet temperature of the mixture. In the experimental results of Kuhn [6], this is in the order of 80 °C. Because of this, the simple models that use a constant wall temperature in modelling only the condenser tube will not be valid. Similarly, a constant heat flux boundary condition is also not a valid approximation. For engineering applications involving vapour condensation in the presence of non-condensable gases, generally both the wall temperature and heat flux at the condenser tube surface are not known a prior and in general only the inlet mass flow rates and the temperatures of the gas mixture and coolant are provided. Because of this, the condenser system including the gas mixture, the condenser wall and the coolant flow needs to be modelled together.

**Fig. 5:**
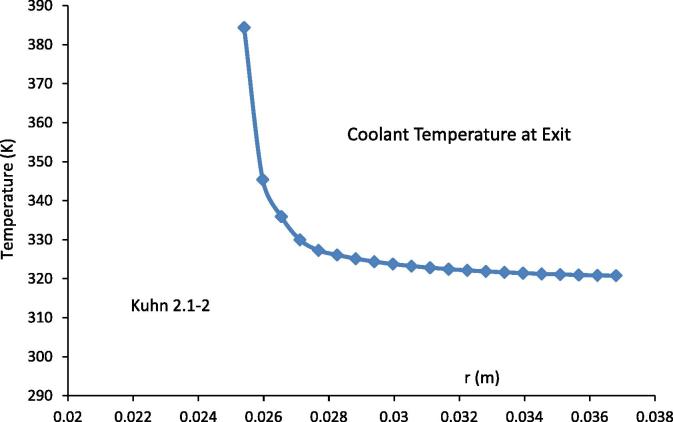
The temperature profile of the coolant at the exit for the experimental conditions of Kuhn 2.1–2 [6].

The results from Figs. 2 (a-f) and 4(a-f) show that, at least near the inlet of the gas mixture region, the difference in the temperature of the condenser wall and that of the adiabatic wall can be quite large as that shown in Fig. 5 for run 2.1–2. This large temperature difference will induce buoyancy forces in the coolant channel due to natural convection. Using the results given in Fig. 5, it can be estimated that the Raleigh number

(24) $$\mathrm{Ra}=\mathrm{Gr}\mathrm{Pr}\approx 9.4\times 10^{6}$$

at the coolant exit. Here $\mathrm{Gr}=\frac{g\beta \Delta T\delta^{3}}{\nu }$ is the Grashof number, $\mathrm{Pr}=\frac{C_{p}\mu }{k}$ is the Prandtl number, β is the volume coefficient of expansion, Δ*T* is the temperature difference between that at the outer surface of the condenser tube and that of the adiabatic wall, and *k* is the thermal conductivity of the coolant. As according to [26], the flow induced by the buoyancy force alone in the annulus cooling channel will be close to turbulent. The estimated heat transfer coefficient [26] due to the buoyancy force will be about 5.7 kW/m^2^K. Because of this, it is recommended that buoyancy forces should be included in modelling the flow and heat transfer in the coolant channel, especially in case of high temperature and high vapour content in the gas mixtures.

**Fig. 6:**
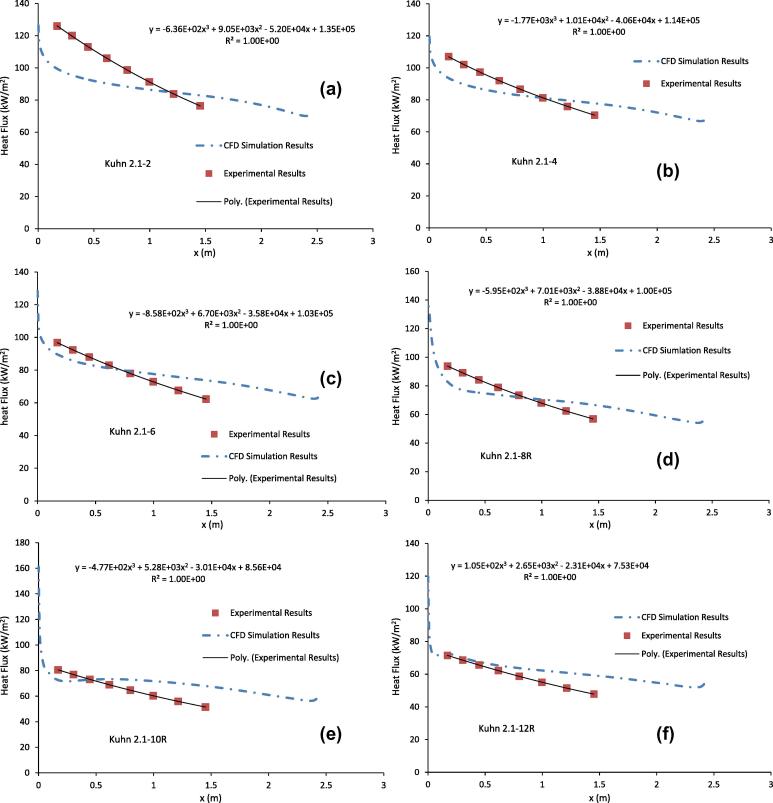
Comparison of heat flux between the CFD simulation results and the derived results from Kuhn [6] for the runs listed in Table 1.

Fig. 6(a-f) show the comparison of the heat flux between the CFD simulation results and the estimated results from Kuhn [6] for the runs listed in Table 1. Kuhn [6] estimated the heat flux using the bulk temperature of the coolant which was in turn estimated using the measured wall temperature of the condenser tube, the wall temperature of the adiabatic wall and the F factor. Kuhn [6] calculated the F factor using the *k*–*ε* turbulence model for the flow and heat transfer in the coolant channel. The heat flux from the CFD simulation was calculated using Eq. (19) and was used as an input boundary condition for CFD simulations of the heat transfer in the condenser tube and coolant jacket. Fig. 6(a-f) show that, although the estimated heat fluxes from the wall temperatures are in the same order of magnitude as those from the CFD simulation results, the trend of the heat fluxes variation with *x* estimated from Kuhn [6] is quite different from that of the CFD simulation results. In Fig. 6(a-f), we also show the 3rd order polynomial curve fitting to the estimated heat fluxes from Kuhn [6] and the curve fitting relationships. It is clear from the curve fittings that the heat fluxes given by Kuhn [6] fit the 3rd order polynomial curves perfectly. On the other hand, the heat fluxes predicted by the CFD simulations show a much more complex variation with *x* and a simple 3rd order polynomial curve fitting is inadequate to represent the heat flux for the vapour condensation in the presence of non-condensable gases.

The heat flux from the CFD simulations as given in Fig. 6(a-f) shows that near the inlet of the gas mixture, the heat flux decreases sharply. This is due to the entrance or developing length effect of the gas mixture flow. The inlet velocity and mass fraction of the water vapour were specified as constants. Different profiles of inlet velocity and mass fraction of water vapour can be used to examine their effects on the heat flux near *x* = 0. The CFD simulation results show that after the initial sharp drop and over much the length of the condenser tube, the heat flux remains fairly constant. This is consistent with the results of centreline temperature as that shown in Fig. 3(a-f) where the decrease in centreline temperature of the gas mixture is not large. Fig. 6(a-f) also show that near the inlet of the coolant, there is a slight increase in the heat flux as *x* increases. This could be due to the entrance effect of the coolant.

The condensation rates as given in Kuhn [6] are not compared with the CFD simulation results since the condensate rates given in Kuhn [6] were derived from the heat flux and it is expected that the results would have the same errors as that of the heat flux.

**Fig. 7:**
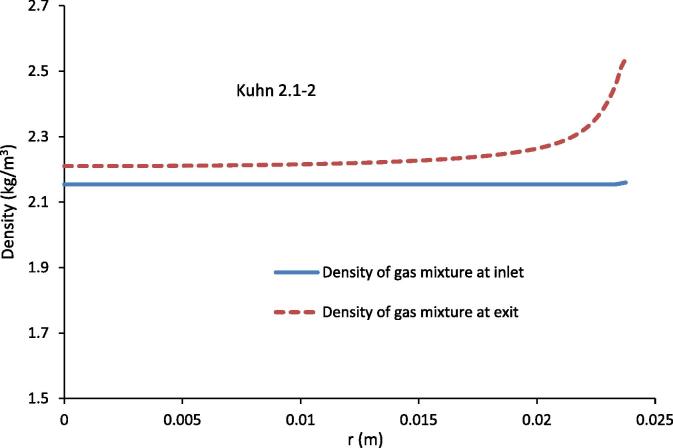
Density of gas mixture at the inlet and exit of the condenser tube for the experimental conditions of Kuhn [6] 2.1–2.

Fig. 7 shows the gas mixture density variation across the condenser tube at the inlet and exit for the experimental conditions of Kuhn [6] 2.1–2. At the inlet, the density as an input is constant, but at the condenser exit, Fig. 6 shows that the density of the gas mixture increases as the condenser wall is approached. This increase in density is due to the decrease of temperature and an increase in the mass fraction of air. Fig. 6 also shows that the average gas mixture density across the tube increases from inlet to exit, again due to both a decrease in average temperature and average mass fraction of water vapour.

**Fig. 8:**
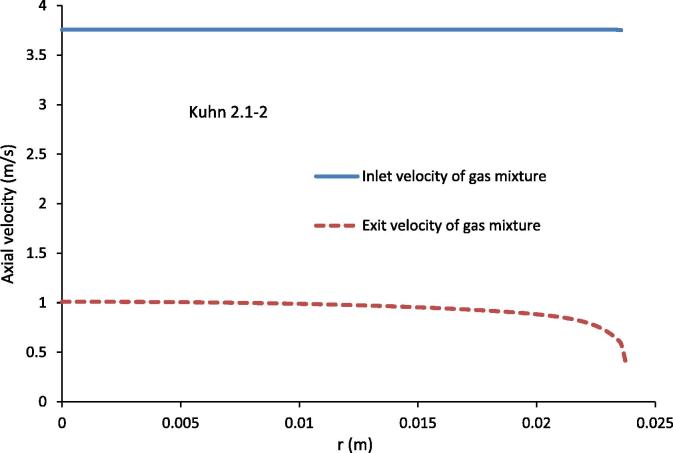
Axial velocity of gas mixture at the inlet and exit of the condenser tube for the experimental conditions of Kuhn [6] 2.1–2.

Fig. 8 shows the axial velocity of the gas mixture at the inlet and exit for the experimental conditions of Kuhn [6] 2.1–2. The inlet velocity of the gas mixture, as an input, is constant across the condenser tube. The axial velocity at the exit shows an almost uniform profile over much of the radius of the tube. The axial velocity at the exit is not zero at the inner surface of the condenser tube. The non-zero velocity at the wall is from the boundary condition for the axial velocity of gas mixture, which was specified using Eq. (15), the surface velocity of the condensate film.

**Fig. 9:**
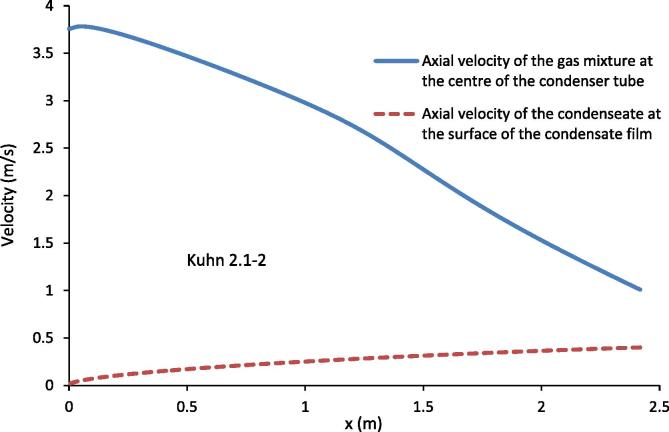
Axial velocity of gas mixture at the centreline of the condenser tube and that of condensate at the condensate film surface for the experimental conditions of Kuhn [6] 2.1–2.

Fig. 9 shows the axial velocity of the gas mixture at the centreline of the condenser tube and that of the condensate at the surface of the condensate film for the experimental conditions of Kuhn [6] 2.1–2. Fig. 9 shows that the axial velocity of the gas mixture at the centreline of the condenser tube decreases rapidly while the axial velocity of the condensate at the interface increase. At the exit of the condenser tube, the velocity of the condensate at the surface of the condensate film is not negligible in comparison with the velocity of the gas mixture. Because of this, it is not appropriate to use stationary wall as velocity boundary condition for the gas mixture.

**Fig. 10:**
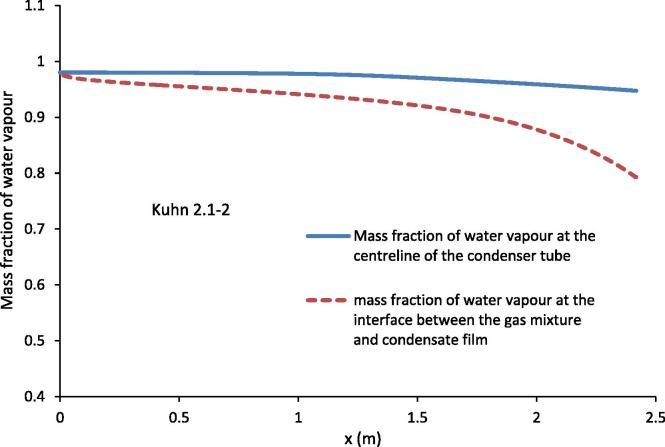
Mass fraction of water vapour at the centreline of the condenser tube and the interface between the gas mixture and condensate film for the experimental conditions of Kuhn [6] 2.1–2.

Fig. 10 shows the variation of the mass fraction of water vapour at the centre of the condenser and that at the interface between the gas mixture and the condensate film. Fig. 10 shows that even through the decrease in the mass fraction of the water vapour is slow at the centre of the condenser tube, the decrease is much faster at the interface. Because of the very high inlet mass fraction (*Y_v_* = 0.98) of the water vapour for run 2.1–2 [6], there is still a quit high average mass fraction of the water vapour at the exit of the condenser tube. As shown in the experimental conditions listed in Table 1, Kuhn [6] increased the mass flow rate of the coolant as the inlet mass fraction of the water vapour was increased. The results in Fig. 10 show that even higher mass flow rate than that listed in Table 1 is required to condense nearly all the water vapour in the condenser for run 2.1–2. This also shows that at the high mass fraction of water vapour and high mass flow rate of the gas mixture, the heat transfer in the coolant may be the limiting factor in the condensation of the water vapour.

## 5 Discussion and conclusions

The condensation of water vapour in the presence of non-condensable gas in a vertical cylindrical tube condenser has been studied using CFD simulation. The CFD simulation for the first time successfully includes the heat and mass transfer in the gas mixture and the heat transfer in the coolant flowing in the annulus channel. Because of this, no assumptions have been made of the wall temperature, heat flux or heat transfer coefficient at the condenser tube wall. Instead, these quantities can be predicted from the CFD simulation.

The CFD simulations of the flow inside the tube condenser of gas mixture were carried out on the gas mixture only. The volume occupied by the condensate film has been neglected. The effect of the condensate film on the gas mixture flow is accounted for through a set of new boundary conditions including the sources for the mass, momentum and energy, a slip boundary condition for the axial velocity and a revised wall temperature taking into account the film thickness. The condensate film thickness is estimated using the Nusselt method by assuming that the shear stresses of both the gas mixture and the condensate match each other at the interface between the gas mixture and the condensate film. The axial velocity of the gas mixture is assumed to match that of the condensate at the interface.

The CFD simulations were conducted using the ANSYS FLUENT®. To overcome the limitations of the FLUENT® in simulating the heat transfer in two separate channels involving multispecies and fluids of different phases, the CFD simulations were carried out asynchronously and iteratively. It is found that this strategy works well. During the CFD simulations, the gas mixture was considered as an ideal gas with thermal properties varying with temperature. The buoyancy forces due to the temperature and mass fraction variations in the gas mixture were taken into account without using the linear approximation while that in the coolant channel was taken into account using the Boussenisq approximation.

The CFD simulation results have been compared with the experimental results of Kuhn [6] for the gas mixture of air and water vapour with inlet mass fractions of water vapour varying from 0.66 to 0.98. The condensation of water vapour for such high vapour content has been considered difficult to predicted using CFD in the past. It is found that the CFD simulation results in general agree well with the measured quantities of Kuhn [6] such as the adiabatic wall temperature, the centreline temperature of the gas mixture and the wall temperature of the condenser tube wall. The simulation results show that for the condensation of high mass fraction of water vapour in the presence of non-condensable gas, the heat transfer in the coolant channel is the limiting factor. The heat flux from the CFD simulations have also been compared with the results from Kuhn [6] who derived these by making several approximations. It is found that the heat flux given in Kuhn [6] can be correlated using a third order curve fitting while the CFD simulation results show a much more complex variation as the vapour is condensed.

The CFD simulation results for the density of the gas mixture, axial velocity and mass fraction of the water vapour across the inlet, outlet and along the condenser tube are presented for the run 2.1–2 of Kuhn [6]. The results are all in agreement with expectations. The results clearly show that the average axial velocity decreases rapidly as water vapour is condensed, the density of the gas mixture increases across the condenser tube and along the condenser and the axial velocity of the gas mixture at the interface between the gas mixture and the condensate film is not small. It is expected that for lower Reynolds numbers of gas mixture at the inlet or long enough condenser tube with high mass flow rate of coolant, the axial velocity of the gas mixture at the interface can be higher than the average axial velocity of the gas mixture.

## Figures and Tables

**Table 1:** Experimental conditions from Kuhn [6].

	*P (kPa)*	*T_in_* (°C)	*W_in_*	${\overset{˙}{m}}_{\mathrm{av}}$ (kg/s)	${\overset{˙}{m}}_{c}$ (kg/s)	*T_c,i_* (°C)
Run 2.1–2	415.3	147.7	0.98	0.01434	0.3521	31.3
Run 2.1–4	390.5	144.8	0.96	0.01464	0.3419	31.0
Run 2.1–6	391.2	142.5	0.92	0.01521	0.3008	30.0
Run 2.1–8R	413.1	144.8	0.85	0.01669	0.2570	27.5
Run 2.1–10R	406.6	140.6	0.76	0.01865	0.2420	26.5
Run 2.1–12R	410.1	135.5	0.66	0.02166	0.2128	25.1

## References

[1] Colburn A.P., Hougen O.A. (1934). Design of cooler condensers for mixtures of vapors with noncondensing gases. Ind. Eng. Chem..

[2] Dehbi A., Guentay S. (1997). A model for the performance of a vertical tube condenser in the presence of non-condensable gases. Nucl. Eng. Des..

[3] Munoz-cobo J.L., Herranz I., Sanchoo J., Tkachenko I., Verlu G. (1996). Turbulent vapor condensation with noncondensable gases in vertical tubes. Int. J. Heat Mass Transfer.

[4] Che D., Da Y., Zhuang Z. (2005). Heat and mass transfer characteristics of simulated high moisture flue gases. Heat Mass Transfer.

[5] M. Siddique, The Effects of Noncondensable Gases on Steam Condensation under Forced Convection Conditions, Ph.D. Thesis, Cambridge, MA, 1992.

[6] S.Z. Kuhn, Investigation of Heat Transfer from Condensing Steam–gas Mixtures and Turbulent Films Flowing Downward Inside a Vertical Tube, Ph.D. Thesis, University of California, Berkeley, 1995.

[7] Kuhn S.Z., Schrock V.E., Peterson P.F. (1997). An investigation of condensation from steam–gas mixtures flowing downward inside a vertical tube. Nucl. Eng. Des..

[8] Li J.D., Saraireh M., Thorpe G. (2011). Condensation of vapor in the presence of non-condensable gas in condensers. Int. J. Heat Mass Transfer.

[9] Revankar S.T., Pollock D. (2005). Laminar film condensation in a vertical tube in the presence of noncondensable gas. Appl. Math. Model..

[10] Rao V.D., Krishna V.M., Sharma K.V., Rao P.V.J.M. (2008). Convective condensation of vapor in the presence of a non-condensable gas of high concentration in laminar flow in a vertical pipe. Int. J. Heat Mass Transfer.

[11] Bucci M., Sharabi M., Ambrosini W., Forgione N., Oriolo F., He S. (2008). Prediction of transpiration effects on heat and mass transfer by different turbulence models. Nucl. Eng. Des..

[12] Benelmir R., Mokraoui S., Souayed A. (2009). Numerical analysis of filmwise condensation in a plate fin-and-tube heat exchanger in presence of non-condensable gas. Heat and Mass Transfer.

[13] Moukalled F., Verma S., Darwish M. (2011). The use of CFD for predicting and optimizing the performance of air conditioning equipment. Int. J. Heat Mass Transfer.

[14] Mimouni S., Mechitoua N., Foissac A. (2011). CFD modelling of wall steam condensation: two phase flow approach versus homogenous flow approach. Sci. Technol. Nucl. Install..

[15] R.Y. Yuann, Condensation from Vapor–gas Mixture for Forced Downflow Inside a Tube, Ph.D. Thesis, University of California, Berkeley, CA, 1993.

[16] Panday J.A. (2003). Two-dimensional turbulent film condensation of vapours flowing inside a vertical tube and between parallel plates: a numerical approach. Int. J. Refrig..

[17] Groff M.K., Ormiston S.J., Soliman H.M. (2007). Numerical solution of film condensation from turbulent flow of vapor–gas mixtures in vertical tubes. Int. J. Heat Mass Transfer.

[18] Laaroussi N., Lauriat G., Desrayaud G. (2009). Effects of variable density for film evaporation on laminar mixed convection in a vertical channel. Int. J. Heat Mass Transfer.

[19] M. Saraireh, G. Thorpe, J.D. Li, Simulation of heat and mass transfer involving vapor condensation in the presence of non-condensable gases in plane channels, in: Proceedings of the ASME/JSME (2011), Eighth Thermal Engineering Joint Conference, AJTEC2011, March 13–17, 2011, Honolulu, Hawaii, USA.

[20] Shih T.H., Lou W.W., Shabbir A., Yang Z., Zhou J. (1995). A new *k*–*e* eddy viscosity model for high Reynolds number turbulent flow-model development and validation. Comput. Fluids.

[21] T. Fujii, Y. Kato, K. Mihara, Expression of transport and thermodynamic properties of air, steam and water, Sei San Ka Gaku Ken Kyu Jo, Report No. 66, Kyu Shu University, KyuShu, Japan, 1977.

[22] Bird R.B., Stewart W.E., Lightfoot E.N. (2007). Transport Phenomena.

[23] Batchelor G.K. (1999). An Introduction to Fluid Dynamics.

[24] ANSYS FLUENT, User’s Guide, Release 13, 2010.

[25] A. Tanrikut, O. Yesin, Experimental research on in-tube condensation in the presence of air, in: Proceedings of a Technical Committee Meeting, IAEA TECDOC-1149, Switzerland, 1998, pp. 14–17.

[26] Holman J.P. (1997). Heat Transfer.

